# It is time to consider third-line options in antiretroviral-experienced paediatric patients?

**DOI:** 10.1186/1758-2652-14-55

**Published:** 2011-11-15

**Authors:** Gert U van Zyl, Helena Rabie, James J Nuttall, Mark F Cotton

**Affiliations:** 1 Division of Medical Virology, Stellenbosch University, and National Health Laboratory Service Tygerberg, Parow, Cape Town, South Africa; 2 Department of Paediatrics and Child Health, Stellenbosch University, and Tygerberg Children's Hospital, Parow, Cape Town, South Africa; 3 Paediatric Infectious Diseases Unit, Red Cross Children's Hospital and University of Cape Town, South Africa; 4 Children's Infectious Diseases Clinical Research Unit, Cape Town, South Africa

## Abstract

**Background:**

The historic use of full-dose ritonavir as part of an unboosted protease inhibitor (PI)-based antiretroviral therapy regimen in some South African children contributes to the frequent accumulation of major PI resistance mutations.

**Methods:**

In order to describe the prevalence of major PI resistance in children failing antiretroviral therapy and to investigate the clinical, immunological and virological outcomes in children with PI resistance, we conducted a cross-sectional study, with a nested case series, following up those children with major PI resistance. The setting was public health sector antiretroviral clinics in the Western Cape province of South Africa, and the subjects were children failing antiretroviral therapy. The following outcome measures were investigated: CD4 count, viral load and resistance mutations.

**Results:**

Fourteen (17%) of 82 patients, referred from tertiary hospitals, had major PI resistance. All these patients were exposed to regimens that included ritonavir as a single PI. Immune reconstitution and clinical benefit were achieved when using a lopinavir/ritonavir-based treatment regimen in these children with prior PI resistance. At first HIV-1 viral load follow up after initial resistance testing (n = 11), only one patient had a viral load of less than 400 copies/ml; at a subsequent follow up (n = 9), the viral loads of five patients were less than 400 copies/ml. Patients retained on LPV/r had lower viral loads than those switched to a non-nucleoside reverse transcriptase inhibitor (NNRTI). However, two of three patients with follow-up resistance tests accumulated additional PI resistance.

**Conclusions:**

In children with pre-existing PI resistance, although initially effective, the long-term durability of a lopinavir/ritonavir-based treatment regimen can be compromised by the accumulation of resistance mutations. Furthermore, a second-line NNRTI regimen is often not durable in these patients. As genotypic resistance testing and third-line treatment regimens are costly and limited in availability, we propose eligibility criteria to identify patients with high risk for resistance and guidance on drug selection for children who would benefit from third-line therapy.

## Background

In South Africa, antiretroviral therapy (ART) became available for adults and children through public sector programmes in 2004. Due to the use of nevirapine (NVP), a non-nucleoside reverse transcriptase inhibitor (NNRTI) for the prevention of mother to child transmission (PMTCT), first-line therapy in children below three years of age includes a protease inhibitor (PI) with two nucleoside reverse transcriptase inhibitors (NRTIs). The preferred PI is lopinavir/ritonavir (LPV/r), which has a high genetic barrier to resistance development.

However, ritonavir (RTV) as a single PI (sPI) was initially used for infants below six months of age and also when rifampicin was needed for co-treatment of tuberculosis, or in some children receiving therapy before the national roll-out guidelines were formulated. The correct dosage for LPV/r in infants below six months of age was established only in 2007 and the boosting of LPV/r with additional RTV when using rifampicin in 2008 [1,2]. Until then, many children were therefore treated with RTV sPI.

We previously documented in children with detectable viral loads that those on RTV sPI were more likely to have major PI resistance than those on LPV/r[3]. We did not observe any mutation accumulation in patients while on LPV/r therapy; however, we had limited follow up of patients with prior PI resistance that had detectable viral loads on LPV/r therapy. We therefore present outcomes in a case series of 14 RTV sPI-exposed children with significant PI resistance to further explore the durability of LPV/r therapy after sPI exposure.

## Methods

We conducted a cross-sectional study, with a nested follow-up case series of those children with major PI resistance. Specimens were received from patients for genotypic resistance testing (GRT) from Tygerberg and Red Cross Children's Hospitals and other antiretroviral treatment sites in the Cape Town Metropole in the Western Cape province of South Africa. Specimens were collected from January 2007 to November 2009.

Patient caregivers (parent or legal guardian) gave written informed consent (and minors assented) as part of an observational study of antiretroviral resistance. Inclusion criteria for GRT were: an available ART history, detectable viral loads (>400 copies/ml, defined by the sensitivity of our test), and recent adequate adherence, as documented by the referring clinician (previous poor adherence did not exclude patients). Viral load testing was with the NucliSens EasyQ system (BioMerieux, Boxtel, The Netherlands). GRT was done by a well-validated "in-house" method for viral RNA, extracted from plasma, followed by reverse transcriptase polymerase chain reaction and bulk automated sequencing [3,4].

Clinical data were recorded in a secure database. Follow-up testing included six-monthly CD4 and viral load counts. We used the geometric mean to indicate central tendency for viral load values, which are not normally distributed. Sequences were imported into the following four resistance interpretation systems (RIS): 1) The Stanford HIVDB v. 6.0.11; 2) Rega v.8.0.2; 3) French ANRS v. 2011.20 accessed through the Stanford Database HIVAlg programme (available from: http://sierra2.stanford.edu/sierra/servlet/JSierra?action=hivalgs), the RegaDB Software, HIV Data Management and Analysis Environment Clinical Edition (available from: http://newbioafrica.mrc.ac.za:8080/regadb-ui/RegaDB); and 4) Geno2Pheno (available from http://www.geno2pheno.org). The Committee for Human Research at Stellenbosch University approved the study.

## Results

### Description of specimens received for genotypic resistance testing

Of 97 specimens, the *protease *gene was sequenced in 88 specimens from 82 patients. The gene could not be amplified or sequenced in nine. Seventy-seven patients had a single specimen each, four patients contributed two specimens each, and one patient had three specimens. We also recorded prior PI therapy and therapy at the time of resistance testing in these patients (Table 1).

**Table 1:** Protease inhibitor exposure and current therapy details of the 82 patients (88 resistance tests)

Prior PI exposure		PI or NNRTI component at the time of resistance testing
PI (n = 53)	LPV/r (n = 30)	LPV/r (n = 30)
	RTV (n = 23)	LPV/r (n = 16)
		LPV/r+ NVP (n = 1)
		EFV (n = 4)
		RTV (n = 2)
No PI (n = 29)		EFV (n = 17)
		NVP (n = 11)
		EFV or NVP at different time points(n = 1)

PI: Protease inhibitor

NNRTI: Non-nucleoside reverse transcriptase inhibitor

Nevirapine: NVP

Efavirenz: EFV

Ritonavir: RTV

At testing, 53 patients had PI exposure. Of these, 47 were treated with PIs (45 with LPV/r and 2 with RTV) and 5 with EFV and 1 with LPV/r plus NVP. Twenty-nine without PI exposure were treated with NNRTIs (17 with EFV, 12 with NVP and one with either NVP or EFV at different time points). Twenty-three patients had received RTV sPI, of whom16 had been switched to LPV/r and one to NVP plus LPV/r. Four were switched to EFV and two remained on RTV at the time specimens were submitted.

### Patients with major PI resistance mutations

Fourteen of 82 patients (17%) had major PI resistance [Genbank: JN087531-JN087547], all of whom were exposed to RTV as a single PI. In these patients, major PI mutations (frequency) were: V33F (n = 3), M46I (n = 4), I54V (n = 13), L76V (n = 2), V82A/M/S (n = 13) and L90M (n = 4). Minor PI mutations were: L10FIV (n = 8), L23I (n = 1), L24I (n = 2), K43T (n = 1) and Q58E (n = 2). Common subtype C polymorphisms were: T74S (n = 3) and L89M (n = 7). When the HIVDB "sensitive", "intermediate" or "resistant" classification is used (which does not include the standard HIVDB "low-level" and "potential low-level" categories), 13 of 14 patients had at least intermediate resistance to LPV/r and atazanvir/ritonavir (ATV/r), five to tipranivir/ritonavir (TPV/r) and only two to darunavir/ritonavir (DRV/r).

In contrast, none of 30 patients treated only with LPV/r as the PI component of their regimens had major PI mutations. Detailed antiretroviral therapy and resistance information for patients with PI resistance are provided in Table 2. The median age of patients with major PI resistance mutations was 36 (interquartile range [IQR]: 43-58) months. The median duration of RTV therapy was 16.8 (IQR: 11.6-26.3) months. For those given subsequent LPV/r therapy, the median duration of LPV/r therapy was 15.9 (IQR: 6.3-39.8) months. Patients who received LPV/r without prior RTV exposure received LPV/r for a median of 14.5 (IQR: 9-24.4) months. At the time of resistance testing, nine were treated with LPV/r, three with RTV, two with efavirenz (EFV) and one with NVP. Six had received NVP as part of PMTCT, two did not receive PMTCT and in six, PMTCT history was unknown.

**Table 2:** Treatment history and resistance in 14 patients with major PI resistance

Study no	Prior Rx	Current Rx	PI resistance	TAMS	LPV/r resistance interpretation				HIVDB: resistance to salvage PIs		
					**Rega**	**HIVDB**	**ANRS**	**Geno2Pheno**	**DRV/r**	**TPV/r**	**ATV/r**

138	AZT, 3TC, RTV	AZT, 3TC, LPV/r	M46I, I54V, V82A, L10F	None	Int	Int	Int	Int	None	None	Int

344	AZT, 3TC, RTV	D4T, ABC, LPV/r	L10V, L24I, K43T, M46I, I54V, T74S, V82A	M41L, D67N, K70R, T215F, K219Q	Res	Int	Res	Res	None	Pot low	Int

345	D4T, 3TC, RTV	AZT, 3TC, EFV	L10I, I54V, V82A	D67N, K70R, T215FY, K219EQ	Int	Int	Int	Int	None	None	Low

87	D4T, 3TC, RTV	AZT, 3TC, RTV	I54V, V82A, L10V, L33F	None	Int	Int	Int	Int	None	Low	Int

34	D4T, 3TC, RTV	D4T, 3TC, RTV	I54V; V82A; L10V	None	Int	Int	Int	Sens	None	None	Low

34b		D4T, 3TC, LPV/r	I54V; L76V; V82A; L10I, A71V	None	Res	Res	Res	Int	Low	None	Low

78	AZT, 3TC, RTV	ABC, DDI, NVP	L33F, I54V, A71V, V82S, L90M	D67N, K70R, K219E	Int	Int	Res	Int	None	Int	Int

78b		AZT, 3TC, LPV/r	L33F, I54V, A71V, V82S, L90M	D67N, K70R, T215I, K219E	Int	Int	Res	Int	None	Int	Int

228	AZT, 3TC, RTV	AZT, DDI, LPV/r	L10V, L23I, M46I, I54V, L76V, V82M	D67N, K70R, T215F, K219E	Res	Int	Res	Res	Low	None	Int

185	D4T, DDI, RTV	AZT, 3TC, EFV	V82A	None	Sens	Int	Sens	Sens	None	None	Low

32	D4T, 3TC, RTV	D4T, 3TC, LPV/r	I54V, V82A, L10I, T74S	None	Int	Int	Res	Int	None	None	Low

32b		D4T, 3TC, LPV/r	L10FI, L33F, I54V, T74S, V82A	None	Int	Int	Res	Int	None	Low	Int

94	D4T, 3TC, RTV	D4T, 3TC, LPV/r	M46I, I54V, V82A, L10F, L24I	None	Int	Int	Res	Res	None	None	Int

29	AZT, 3TC, RTV	AZT, 3TC, LPV/r	I54V	None	Sens	Sens	Sens	Sens	None	None	Pot low

38	D4T, DDI, RTV	D4T, 3TC, LPV/r	I54V; V82A; L90M; Q58E; A71V	None	Int	Int	Res	Int	None	Low	Int

324	D4T, 3TC, RTV	D4T, 3TC, LPV/r	M46V, I54V, A71V, T74S,V82A, L90M	None	Int	Int	Res	Res	None	None	Int

49	D4T, DDI, RTV	D4T, DDI, RTV	I54V; V82A; L90M; L33F; Q58E	M41L; D67N; T215Y	Int	Int	Int	Sens	None	Low	Int

**Rega: **Rega v.8.0.2 resistance interpretation system

**HIVDB: **The Stanford HIV Data Base v. 6.0.11 resistance interpretation system

**ANRS: **The French ANRS v. 2011.20 resistance interpretation system

**Geno2Pheno: **The Geno2Pheno resistance interpretation system

For comparison of resistance interpretation systems, the "Sensitive, Intermediate and Resistant" (SIR) classification was used: **Sens: **Sensitive (high-level resistant), **Int: **Intermediate-level resistance, and **Res: **Resistant

The standard HIVDB classification includes additional categories: **Low: **Low-level resistance, **Pot low: **Potential low-level resistance

**Prior Rx: **Prior treatment

**Current Rx: **Treatment at the time of resistance test

**TAMS: **Thymidine-associated mutations

*All patients receiving 3TC at the time of resistance testing had the M184V mutation*.

*All patients included in the table had HIV-1 genotype C pol sequences according to the Rega HIV-1 subtyping tool*.

*Specimens labelled "b" are repeat specimens on the same patient*.

The *M184V *mutation, conferring resistance to lamivudine (3TC), was detected in all 14 specimens from 11 patients failing 3TC-containing therapy with major PI resistance. At the time of resistance testing, six of 12 with viral load data had values between 1000 and 5000 (5000 is the current recommendation for therapy switching in South Africa). Five patients had three or more thymidine analogue mutations (TAMs), in addition to significant PI mutations. Four of the 14 had intermediate to high-level EFV and NVP resistance.

Eleven patients were followed up after initial resistance testing. Initially, only one suppressed to less than 400 copies/ml. Subsequently, five of nine suppressed to less than 400 copies/ml (Table 3). Only two of five children continuing on an NNRTI regimen had TAMs, but none had virological suppression and were therefore at high risk for developing NNRTI resistance.

**Table 3:** Follow up CD4% and HIV-1 viral load after detecting major PI

Patients who continued on an NNRTI regimen	CD4% range (mean CD4%); number (n) of patients	HIV-1 VL range (geometric mean of detectableVL) in IU/ml; number (n) of patients
Baseline*	19.9 - 49.6 (32.2); n = 5	2300-47,000 (15,804); n = 5

First follow up	23.2-39.7 (30.8); n = 5	9400-25,000 (14,397); n = 5

Second follow up	22.9-46.4 (32.4); n = 5	700-38,000 (5504); n = 4

**Patients who continued on an LPV/r regimen**		

Baseline*	29.4-39.9 (34.6); n = 5	1000-34,000 (3898); n = 6

First follow up	25.6-36.6 (32.4); n = 5	1 × LDL, remainder 1800-38,000 (7473); n = 6

Second follow up	27.2-40.8 (35.6); n = 5	1 × LDL, remainder 320-390 (355); n = 5

**Total: All patients**		

Baseline*	17.1-49.6 (31.9); n = 11	1000-47,000 (6450); n = 12

First follow up	23.2-39.7 (31.4); n = 11	1 × LDL, remainder 1800-38,000 (9558); n = 11

Second follow up	22.9-46.4 (33.1); n = 11	1 × LDL, remainder 320-38,000 (1794); n = 9

VL: Viral load

*Baseline: Refers to values at the time of the first resistance test that resulted in a decision to either continue a PI regimen or switch to an NNRTI regimen

First follow up: Approximately six months after baseline

Second follow up: Approximately 12 months after baseline

LDL: Lower than detection limit (of 357 copies/ml)

Three of six patients who continued on LPV/r had TAMs, Nevertheless, patients who continued on LPV/r achieved lower viral loads than those on an NNRTI. Of the 11 patients followed up, four patients had three-class (PI, NRTI and NNRTI) resistance compromising future therapy options.

Three patients (all with at least intermediate resistance to LPV/r according to Stanford interpretation) had follow-up resistance tests: in two, three additional PI mutations accumulated over 379 days (patient 34) and two additional PI mutations in 498 days (patient 32) while treated with LPV/r. The third patient (patient 78) accumulated no additional PI mutations in a year, between tests, receiving an NVP- based regimen for the first month and an LPV/r regimen for the remainder. Despite acquiring an additional *TAM*, he maintained low-level viraemia, the CD4 percentage improving from 19.9% to 37.6%. Therapeutic drug monitoring found that LPV levels were inadequate. After improved adherence and dose adjustment, the patient had one undetectable viral load, later followed by a recurrence of failure. Apart from pre-existing neuro-developmental delay, he developed no new HIV-related conditions.

Table 3 summarizes the plasma HIV RNA levels and CD4 percentage follow-up tests after resistance testing, categorized by whether patients continued on an NNRTI regimen (EFV) or LPV/r.

## Discussion

### Monitoring and treatment in South Africa

Responding to antiretroviral failure and selecting an optimal regimen is very context specific. In South Africa, viral load monitoring is routinely available, but with only very limited access to antiretroviral resistance testing and with only two lines of therapy for children. In infants exposed to nevirapine, through a PMTCT intervention, there may effectively be only one regimen. In young sub-Saharan African children, who often start therapy in infancy, an NNRTI regimen seems less likely to suppress viral loads than a boosted PI regimen [5], and in a recent multicentre study, P1060, an NVP regimen has been shown to be inferior to an LPV/r regimen both in children exposed to NVP through PMTCT [6] and in those unexposed [7].

Furthermore, children who were switched, when virologically suppressed from LPV/r to NVP were more likely to have viral loads of more than 1000 copies per ml and harbour resistance than those retained on LPV/r [8]. This is in contrast to the multi-centre PENPACT-1 study where outcomes for PI and NNRTI regimens were similar; however, the median age was 6.5 years (much older than in P1060), and in 48%, the PI prescribed was nelfinavir, which has a lower genetic barrier than LPV/r.

### Detected protease inhibitor resistance

We found that 14 out of 23 children with historic exposure to a regimen that included RTV sPI had major PI resistance, whereas none of 30 given LPV/r had major PI resistance. Nevertheless, it is not known if RTV sPI exposure *per se *was aetiological in selecting for PI resistance, in all cases, as other factors, such as longer therapy duration[3] and concomitant rifampicin use, could have contributed to PI resistance. The high prevalence of major PI resistance mutations (14 out of 82 or 17%) in this study cannot be extrapolated to the population as a whole as most of these specimens were referred from tertiary hospitals. However, this may represent a typical setting, which takes care of paediatric patients with long-term failure.

As we did not observe any PI resistance, despite having detectable viral loads, in nine of the 23 patients treated with LPV/r and prior RTV sPI treatment, and 30 out of 30 without prior RTV sPI, their virological failure was most likely due to poor adherence or inadequate dosage. This concurs with a French study that found a very low rate of PI resistance in children initiated on LPV/r despite a high prevalence of virologic failure [9]. Three patients (patients 32, 334 and 344) harboured T74S, a common HIV-1 subtype C *protease *polymorphism, which is found in higher frequencies in patients treated with PIs, especially nelfinavir. It has been reported to possibly restore fitness in patients with multiple PI resistance and to increase susceptibility to ritonavir and indinavir [10].

### Detected NRTI resistance mutations

Once major PI resistance was present, as expected, all children on lamivudine (3TC) had the M184V mutation. 3TC has a low genetic barrier and M184V occurs early during true drug failure [11,12]. A high prevalence of M184V has been reported in other studies in children from sub-Saharan Africa [13-15]. Nevertheless, 3TC is still preferred as a component of first-line therapy and often retained in second-line regimens for the following reasons: it has excellent tolerability and M184V increases susceptibility towards other NRTI components, such as AZT, D4T or tenofovir (TDF). Furthermore it reduces viral fitness, slows the accumulation of TAMs [12,16,17] and may have clinical and immunological benefit [18].

### Outcomes after detecting PI resistance

Patients who continued on an LPV/r regimen had a better virological response than those switched to an NNRTI regimen. However, in two of three patients, who were switched to LPV/r after RTV sPI, additional mutations were observed in their second specimens, increasing PI resistance, thus questioning the durability of LPV/r therapy. However, despite significant PI resistance in 12, with additional TAMs in five children, there was no immunologic deterioration and viral loads remained relatively low in the majority.

This may be due to residual efficacy of the antiretroviral drugs (especially LPV/r), especially at increased plasma levels [19] and the reduced fitness (crippling effect) of some resistance mutations, such as *M184V*, and some PI resistance mutations. Nevertheless, children require ART for life. Inadequate response to therapy may have developmental and neurological consequences and could seriously compromise quality of life. Non-suppressive antiretroviral therapy may in the long run compromise future therapy options through the accumulation of resistance mutations, despite intermediate-term immunological and clinical benefits.

### Criteria for genotypic resistance testing

The use of RTV sPI in children contributed to a cohort with an increased risk of PI resistance and therapy failure [20]. Although children who never received an unboosted PI may also develop PI resistance, the current risk is probably too low to include this in criteria for genotypic resistance testing for resource-limited settings. A good adherence history, in combination with random LPV plasma concentration measurement (which cost only about US$40 in the South African state sector), may exclude patients with very poor adherence from unnecessary GRT, which is more expensive (about US$300 for in-house testing through the National Health Laboratory Service, the public laboratory service provider in South Africa).

An adequate plasma lopinavir concentration does not exclude periods of poor adherence as ingesting few doses before phlebotomy could result in adequate concentrations. However, a low level is indicative of poor adherence. Such random testing has been shown to be valuable in South African adults on second-line therapy [21]. A further benefit of LPV plasma level monitoring is to facilitate dose adjustment and achieve virological suppression. An additional surrogate for adherence is macrocytosis in patients on either zidovudine or stavudine [22,23].

Due to the reduced fitness of some resistant viruses, a high percentage of children with PI resistance may have relative low viral loads, as seen by the number with viral loads below 5000 copies/ml. Therefore, 1000 copies/ml may be an appropriate cut off for resistance testing in patients with a high pre-test probability of protease resistance (such as prior exposure to an RTV sPI ART regimen). Therefore, we recommend using 1000 copies/ml, rather than 5000 copies, as suggested by the World Health Organization, for those who might benefit from resistance testing.

### Defining criteria for third-line therapy

Children who are switched to an NNRTI regimen at the time of PI failure are likely to have an increased risk of failure and resistance, due to the low genetic barrier of the regimen, previous exposure to NVP for PMTCT, and probable sub-optimal adherence. Furthermore, those failing a PI regimen, but with NRTI resistance (such as TAMs) are unlikely to achieve full virological suppression on a second-line NNRTI regimen, and thus rapidly acquire NNRTI resistance. There is therefore a need for a durable third-line combination. However, third-line therapy for children not responding to the currently available regimens is more costly than standard first- or second-line therapy. Therefore, defining indications for third-line therapy in a cost-effective manner is essential.

Two candidate PIs for salvage are TPV/r and DRV/r. Proposed criteria for GRT and third-line ART regimens in children in a resource-limited setting are provided here:

### Criteria for paediatric genotype resistance testing (GRT)

#### Criteria A and B must be met

A) Failure of LPV/r regimen (two sequential viral loads >1000 copies/ml, despite confirmed adherence of >90%) while LPV drug levels are within the therapeutic range.

B) Prior exposure to an unboosted sPI regimen, such as RTV.

### Criteria for third-line ART regimens following GRT

#### Criteria A AND (B OR C) must be met

A) Susceptible or low-level resistance to the proposed high barrier salvage PI (DRV/r or TPV/r) by the Stanford HIVDB RIS.

B) Intermediate resistance to LPV/r (including either Rega, HIVDB, ANRS or Geno2Pheno) with three or more TAMs.

C) High-level LPV/r resistance (including either Rega, HIVDB, ANRS or Geno2Pheno).

### Selecting a third-line regimen

Although continuing therapy without virological suppression is likely to result in additional resistance accumulation, stopping antiretroviral therapy causes rapid clinical and immunological deterioration [24]. In selecting an optimal third-line regimen, a balance between tolerability, residual activity, fitness benefit and the resistance threshold of a regimen should be sought. Where possible, there should be at least good susceptibility (no more than low-level resistance) to two of a three-drug regimen. The most essential component is a PI with a high barrier to resistance, such as DRV/r or TPV/r. The choice is guided by the resistance pattern and the age of the patient. TPV/r is available in a liquid formulation for children as young as two years, whereas DRV/r is only available in tablets for children older than six years. DRV has a better side-effect profile than TPV.

Therapy history and genotypic resistance testing should guide the choice of the best NRTI backbone. When the selective pressure of a particular drug is removed, resistance may become undetectable, but remains clinically relevant. Almost all who previously failed a regimen including 3TC have the *M184V *mutation irrespective of the current genotypic result. TAMs and other NRTI resistance mutations, such as *K65R, L74V *and multiple NRTI resistance mutations, are especially valuable in determining the best NRTI backbone. Quite often, in the presence of TAMs, tenofovir (TDF) is the only NRTI with full susceptibility. However, there is no formulation for patients who weigh less than 30kg. Didanosine (DDI) often shows susceptibility. However, its poor tolerability could contribute to a high failure rate.

Even when the genotype suggests combining DDI and abacavir (ABC), one should consider that resistance to both drugs are conferred by the same mutations (*L74V *and *K65R*), thus potentiating rapid failure. Rarely, susceptibility to all NRTIs is lost, necessitating the use of other drugs classes in combination with DRV/r or TPV/r. 3TC could be retained despite resistance, as we have discussed. Other valuable salvage drugs, such as the integrase inhibitor, raltegravir, the second-generation NNRTI, etravirine, and the CCR5 inhibitor, maraviroc, are not yet licensed for children.

Recent data has shown that raltegravir is valuable in paediatric treatment [25]. When raltegravir is used in treatment-experienced children, due to its low genetic barrier, the proposed regimen must be able to achieve full virological suppression. It should therefore combine potent and high genetic barrier drugs, such as DRV/r or TPV/r. The same applies to etravirine, especially with prior exposure to NNRTIs. However, the addition of raltegravir or etravirine may double the cost of the regimen. A practical approach is to combine a high-barrier PI (DRV/r or TPV/r) with at least one other drug with full sensitivity and a third with some beneficial effect (such as 3TC). Expedited viral load testing should occur within two to three months.

Successful third-line therapy of paediatric patients is hindered by the lack of paediatric formulations and high costs, with dosing especially problematic for children younger than six years, largely a result of the low priority that is given globally to the development of paediatric formulations and regimens [26]. 3TC monotherapy and other sub-optimal interim measures, although being used in some resource-limited settings, are not evidence based, whereas continued PI therapy, even when it does not achieve virological success, could nevertheless render immunological and clinical benefit in children [27], but at the potential cost of resistance accumulation.

## Conclusions

The historic use of an unboosted PI regimen contributed to a cohort of children at increased risk of having compromised first- and second-line antiretroviral regimens. Therefore, there is an urgent need for affordable access to third-line drugs for children in lower- to middle-income countries. Furthermore, there is a need to develop criteria to identify those in whom genotypic antiretroviral resistance testing could assist in decision making.

## Competing interests

The authors declare that they have no competing interests.

## Authors' contributions

GUvZ wrote the draft manuscript. MFC provided oversight over the study. JJN and HR assisted with patient selection and recruitment. MFC, JJN and HR assisted with writing, contextualizing and revising the final manuscript. All authors approved the final manuscript.
